# Evaluation of anatomical and physiological traits of *Solanum pennellii* Cor. associated with plant yield in tomato plants under water-limited conditions

**DOI:** 10.1038/s41598-020-73004-4

**Published:** 2020-09-29

**Authors:** Françoise Dalprá Dariva, Mariane Gonçalves Ferreira Copati, Herika Paula Pessoa, Flávia Maria Alves, Felipe de Oliveira Dias, Edgard Augusto de Toledo Picoli, Fernando França da Cunha, Carlos Nick

**Affiliations:** 1 Departamento de Agronomia. Programa de Pós-graduação em Fitotecnia, Universidade Federal de Viçosa, Av. P.H. Rolfs, s/n, Campus Universitário, Viçosa, MG 36570-900 Brazil; 2 Departamento de Biologia Vegetal. Programa de Pós-graduação em Fitotecnia, Universidade Federal de Viçosa, Av. P.H. Rolfs, s/n, Campus Universitário, Viçosa, MG 36570-900 Brazil; 3 Departamento de Engenharia Agrícola. Programa de Pós-graduação em Engenharia Agrícola, Universidade Federal de Viçosa, Av. P.H. Rolfs, s/n, Campus Universitário, Viçosa, MG 36570-900 Brazil

**Keywords:** Plant sciences, Plant breeding

## Abstract

Although intensively studied, few works had looked into *S. pennellii*’s ability to cope with water-deficit conditions from a breeding point of view. In this study, we assessed potential traits of *S. pennellii*, that had previously been linked to high yields in other plant species, under long-term water-limited conditions and made a parallel with plant yield. For this purpose, the drought-resistant tomato genotypes IL 3–5 and IL 10–1, and the drought-sensitive IL 2–5 and IL 7–1 at seed level, together with both parents the *S. pennellii* accession LA 716 and the cultivar M82 were kept at 50 and 100% ASW throughout the growing season. Our findings confirm the superiority of LA 716 under water-limited conditions compared to the other *S. lycopersicum* genotypes in terms of plant water status maintenance. Percentual reduction on plant yield was higher in IL 3–5 and IL 10–1 than in M82 plants, indicating no correlation between drought resistance on germination and plant productive stages. A strong positive correlation was found between fruit yield and A, g_s_, and Ψ_leaf_ at 50% ASW, suggesting these traits as important selection criteria. LT and g_min_, LA 716’s most promising traits, did not show a linear correlation with fruit yield under low water regimes. This study unravels traits behind tomato performance under water-limited conditions and should work as guidance for breeders aiming at developing drought-resistant tomato cultivars.

## Introduction

Tomato (*Solanum lycopersicum L.*) is the second-largest vegetable crop grown and consumed worldwide. According to FAO’s database, a total of 181 million tonnes of tomatoes were grown in 2018, which occupied almost 5 million hectares of farmland (https://www.fao.org/faostat). Tomato’s great popularity is associated not only with its taste, pleasant flavor and versatility in its use but also to its nutritional value. Tomato fruits contain vitamins, minerals^1^, and high levels of antioxidant substances (carotenes, especially lycopene) whose consumption has been linked to reduced incidence of cardiovascular diseases and some types of cancer^2,3^.

High yields observed today in modern cultivars rely on the consumption of large amounts of water by crops throughout the whole growing cycle. This high-water demand by crops is alarming since it restricts food production to areas where rainfall is abundant and well-distributed in case of non-irrigated farming, or increases production costs significantly in irrigated farmland^4^. Recent changes in climate have led to reduced water availability for agriculture in some regions of the world^5,6^, which makes it impracticable to grow high water requirement crops such as tomatoes on these locations. Therefore, in this current scenario of water insecurity, developing high-yield less water-demanding cultivars through plant breeding is considered to be the most promising strategy.

Plant breeding programs are based on finding favorable alleles for the trait of interest and transferring these alleles to elite germplasm. In tomato, drought resistance is observed in wild species, especially *Solanum pennellii* Cor^7–11^.

Because of its origin and evolution in the western slopes on the Andes in central Peru, an extremely dry region^9^, *S. pennellii* exhibits several adaptative mechanisms that ensure its survival in arid environments^10^. *S. pennellii* plants are able to sustain tissue hydration longer than *S. lycopersicum* plants after water suspension^9^. Since *S. pennellii* has a shallow, poor-developed root system^12^, its capacity to maintain high water status in arid environments is probably due to morphophysiological and anatomical modifications in the aerial part of the plant, including cuticular composition associated with increased resistance to water flux^13^, smaller leaf surface area, greater leaf thickness, and stomatal behavior that conserves water^14^. A complete study and comprehension of such modifications, triggered or not by low soil water availability, as well as its correlation with plant yield, is the first and most crucial step of a tomato breeding program for drought resistance and the primary goal of this study.

To dissect drought resistance conferred by *S. pennellii,* Eshed and Zamir^15^ developed an introgression line population currently composed by 76 lineages (ILs)^16^. This population was formed by initially crossing the processing tomato cultivar M82 with the *S. pennellii* wild accession LA 716. The next generation was then subjected to successive cycles of backcrossing and marker-assisted selection (MAS), with M82 as the recipient parent, so that each IL is genetically identical to M82 except for a single homozygous chromosome segment donated by the wild parent (*S. pennellii*)*.* Therefore, all genetic variation observed between an IL and the cultivar M82 is attributed to the introgressed segment that it holds. Together all ILs provide total coverage of *S. pennellii*’s genome. Therefore, it is possible to identify all chromosome segments of *S. pennellii* associated with its drought resistance by exposing all 76 ILs to drought stress conditions. Once identified, it is easier to transfer this resistance into elite plant materials through MAS.

The *S. pennellii* IL population has been extensively studied and it is now considered a model population when it comes to understanding the genetic architecture of several complex traits in tomato plants, including yield^15,17^, fruit quality^15,17–21^, photosynthesis^22^, leaf morphology^23^, and leaf anatomy^24^. The majority of these studies were, however, performed under well-watered conditions, which means that little is known about QTL expression under water deficit. Besides, none of them have taken into account how morphological and anatomical traits of *S. pennellii* affects plant performance under drought conditions, especially in terms of fruit yield maintenance.

By reviewing the literature available on *S. pennellii,* we found that the increased leaf thickness and reduced minimum epidermal conductance to water vapor previously reported for this species^13,24^ are candidate traits for tomato improvement for drought resistance as they are commonly present on high-yielding genotypes of several crop species. In order to study how these traits are affected by low soil water availability, and if they indeed contribute to maintaining fruit yield under water-limited supply, four tomato introgression lines (IL 2–5, IL 3–5, IL 7–1 and IL 10–1) together with the parents M82 and *S. pennellii* accession LA 716, were grown under 50 (stress) and 100% (control) available soil water (ASW) throughout the entire growing season. The ILs were selected based on their level of resistance to drought on germination and early seedling growth stages, in which IL 3–5 and IL 10–1 displayed the highest level of drought resistance and IL 7–1 and IL 2–5 the lowest (Supplementary Fig. 1.). Assuming the fact that to be considered drought-resistant a tomato genotype must exhibit a fair level of drought resistance on all developmental stages of plant growth, our results will also provide additional information on whether or not IL 3–5 and IL 10–1 should move on to the next phase of our tomato breeding program. A full analysis of plant physiological behavior, which has serious implications on total plant productivity, was also possible as we worked with a restricted number of genotypes. As important yield-associated traits were assessed under limited water supply, we believe that our findings will serve as guidance for breeders aiming at developing drought-resistant tomato cultivars.

**Figure 1:**
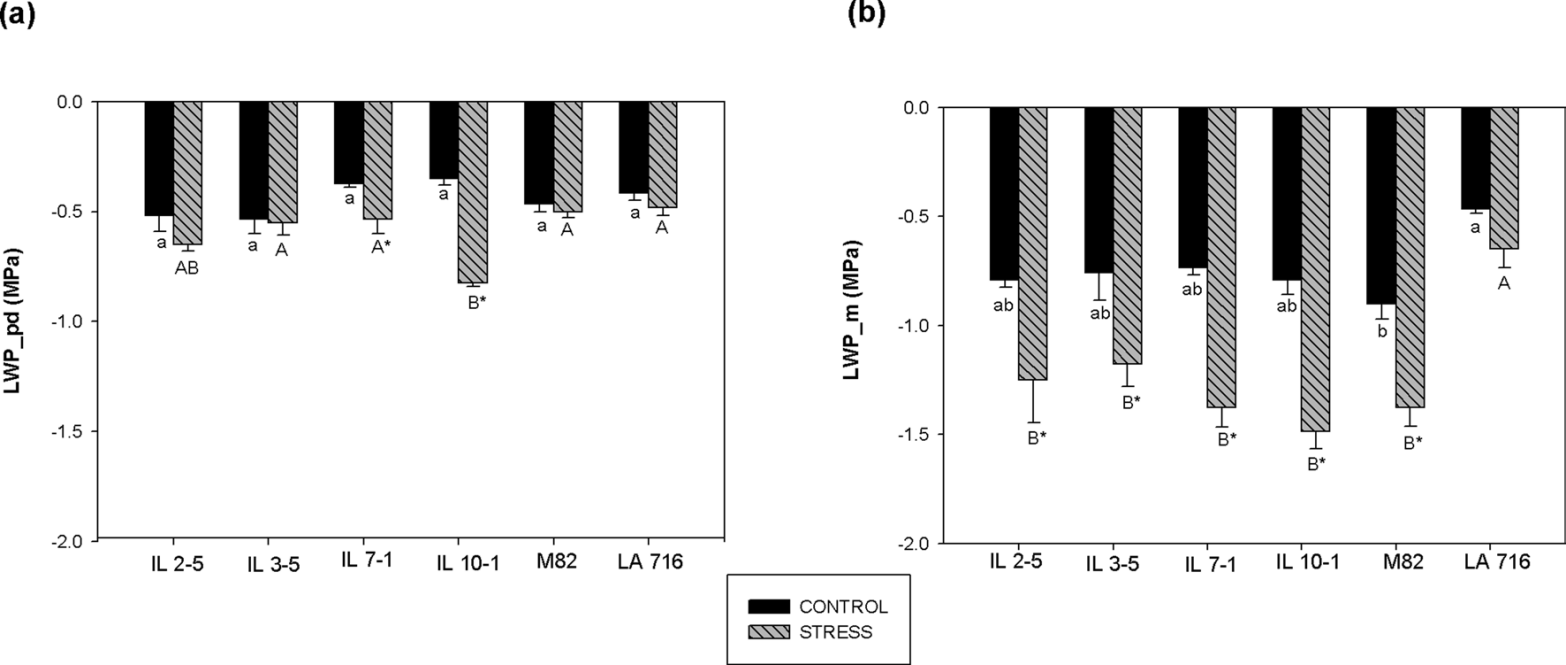
Leaf water potential at pre-dawn (03:00–05:00) (LWP_pd) (**a**) and midday (12:00–14:00) (LWP_m) evaluations (**b**) of tomato genotypes grown under two different water regimes (50 and 100% ASW), quantified on top third leaves, 60 days after the beginning of the stress treatment. Same lower- and upper-case letters indicate that the genotypes did not differ by the Tukey test (*p* > 0.05), in the control (100% ASW), and in the stress treatment (50% ASW), respectively. The asterisk (*) indicates statistical difference between control and stress treatment for the same genotype. Data are expressed as means ± standard error.

## Results

### Water deficit stress

Figure 1 shows leaf water potential (Ψ_leaf_ ) mean values observed for the studied genotypes on both water regimes. At the pre-dawn evaluation, reduction in Ψ_leaf_ caused by the low water regime was observed only for IL 7–1 and IL 10–1 (*p* < 0.05) (Fig. 1a). Ψ_leaf_ between genotypes did not differ at 100% ASW (*p* > 0.05) (Fig. 1a). At midday evaluation, the low water regime reduced Ψ_leaf_ of all genotypes except LA 716 (Fig. 1b). Ψ_leaf_ mean values observed for *S. lycopersicum* genotypes at 50% ASW varied from − 1.18 (IL 3–5) to − 1.48 MPa (IL 10–1) and they were on average 0.5 MPa lower than those observed at 100% ASW. LA 716, known for its drought resistance, showed high Ψ_leaf_ on both water regimes (− 0.47 and − 0.65 MPa on average at 100 and 50% ASW, respectively), differing from the other genotypes by the Tukey’s test (*p* < 0.05) (Fig. 1b). Ψ_leaf_ of LA 716 was similar on both pre-dawn and midday evaluations (an average of − 0.42 and − 0.47 MPa at 100% ASW, and − 0.48 and − 0.65 MPa at 50% ASW, respectively). Note that the ILs behavior at midday evaluation did not differ from the sensitive genotype M82 (*p* > 0.05).

### Physiological traits

**Figure 2:**
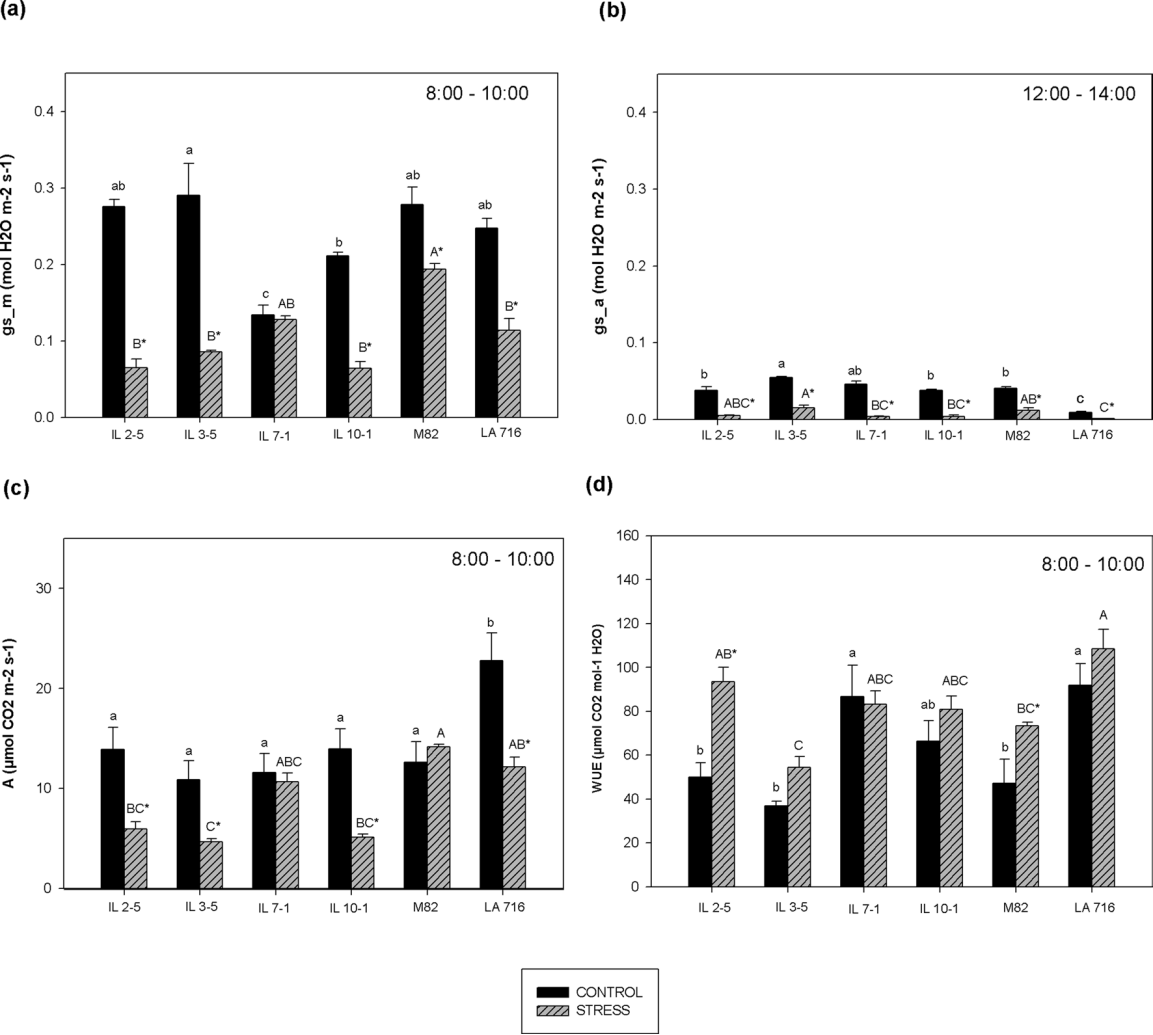
Physiological traits stomatal conductance in the morning (8:00–10:00) (gs_m) (**a**) and in the afternoon period (12:00–14:00) (gs_a) (**b**), photosynthesis (*A*) (**c**), and water use efficiency (WUE) (**d**) of tomato genotypes grown under two different water regimes (50 and 100% ASW), quantified on top third leaves, 60 days after the beginning of the stress treatment. *A* was recorded only in the morning period. WUE was expressed as *A*/g_s_ ratio in the morning period. Same lower- and upper-case letters indicate that the genotypes did not differ by the Tukey’s test (*p* > 0.05), in the control (100% ASW), and in the stress treatment (50% ASW), respectively. The asterisk (*) indicates statistical difference between control and stress treatment for the same genotype. Data are expressed as means ± standard error.

During morning evaluations and under optimum irrigation (100% ASW), LA 716 showed high stomatal conductance (g_s_), statistically identical to g_s_ values observed for M82. Under 50% ASW, g_s_ was lower for all the studied genotypes except IL 7–1 (Fig. 2a).

In order to conserve water at critical days hours (12:00–14:00), all genotypes from both water regimes showed a substantial decrease in g_s_ (Fig. 2b). The lowest g_s_ mean values were observed for LA 716, on both water regimes. The progenitors LA716 and M82 differed stomatal conductance values in water deficit treatments, independently from the morning or afternoon evaluation period (*p* < 0.05) (Fig. 2b). On the other hand, there were significant differences among the ILs, although they could not be distinguished based on g_s_, according to their expected resistance to water deficit (Fig. 2b).

LA 716 showed surprisingly high values of photosynthesis (*A*) when grown under optimum watering conditions (22.78 µmol CO_2_ m^−2^ s^−1^ on average while in M82 *A* mean value was 14.61 µmol CO_2_ m^−2^ s^−1^) and it was statistically different from the other genotypes (*p* < 0.05). However, *A* values of LA 716 and M82 did not differ at 50% ASW (Fig. 2c). The water regime affected all genotypes towards reduction in *A* except IL 7–1 and M82. *A* of IL 3–5 and IL 10–1, both considered drought-resistant on a previous study, together with IL 2–5, considered drought-sensitive, was greatly affected by the low water regime (Fig. 2c).

Genotype × water regime interaction was non-significant for the variables intercellular CO_2_ concentration (Ci), leaf temperature (T_leaf_), and transpiration rate (*E*) (*p* > 0.05). Ci and *E* reduced as affected by the low soil water content on the stress treatment, whereas T_leaf_ increased (*p* < 0.05) (Supplementary Table 1). Significant differences between genotypes were observed only for Ci (Supplementary Table 1). Ci, *E,* and T_leaf_ mean values observed at 100% ASW were 290.04 µmol CO_2_ mol^−1^, 5.09 mmol H_2_O m^−2^ s^−1^ and 26.94 °C whereas at 50% ASW they were 247.69 µmol CO_2_ mol^−1^, 2.51 mmol H_2_O m^−2^ s^−1^ and 27.86 °C, respectively.

**Table 1:** Number of fruits per plant (FN), average fruit diameter (FD), average fruit length (FL), and fruit fresh weight (FW) of five tomato genotypes (IL 3–5 and IL 10–1, considered drought-tolerant, IL 2–5 and IL 7–1, considered drought-sensitive on a previous study, and the processing tomato cultivar M82) kept at two different irrigation regimes (50 and 100% ASW) throughout the entire growing season. Means ± standard error followed by the same lower-case letters within each column (vertical comparison) and the same upper-case letters within each row (horizontal comparison) indicate that no statistical difference was found by the Tukey’s test at 0.05 significance level.

Genotype	Number of fruits per plant				Fruit diameter (cm)			
	Control		Stress		Control		Stress	
IL 2–5	219.22 ± 25.03	abA	165.89 ± 30.13	abA	27.27 ± 1.13	dA	19.11 ± 1.61	bB
IL 3–5	137.22 ± 1.66	bcA	114.33 ± 14.22	abA	35.04 ± 0.76	bcA	20.85 ± 0.70	bB
IL 7–1	118.78 ± 14.13	cA	160.11 ± 33.62	abA	36.55 ± 0.88	abA	20.32 ± 1.59	bB
IL 10–1	250.67 ± 36.57	aA	182 ± 14.26	abB	30.34 ± 2.85	cdA	16.03 ± 0.72	bB
M82	83.56 ± 2.63	cA	74.56 ± 3.47	bA	41.78 ± 1.00	aA	27.59 ± 0.72	aB

Genotype	Fruit length (cm)				Fruit fresh weight (Kg plant^−1^)			
	Control		Stress		Control		Stress	
IL 2–5	33.74 ± 1.85	bA	25.32 ± 2.70	bB	1.95 ± 0.15	bcA	0.49 ± 0.05	aB
IL 3–5	43.76 ± 2.25	aA	26.11 ± 0.95	abB	2.66 ± 0.15	aA	0.56 ± 0.02	aB
IL 7–1	46.11 ± 1.00	aA	26.10 ± 2.31	abB	2.20 ± 0.13	bA	0.56 ± 0.03	aB
IL 10–1	26.21 ± 2.49	bA	16.98 ± 1.32	cB	1.59 ± 0.06	cA	0.35 ± 0.02	aB
M82	49.92 ± 0.32	aA	33.32 ± 0.70	aB	2.14 ± 0.03	bA	0.72 ± 0.01	aB

Plants grown at 50% ASW showed water-use efficiency (WUE) lower than plants grown under 100% ASW (*p* < 0.05), probably due to a decrease in g_s_. The highest WUE was observed for LA 716 (108.43 µmol CO_2_ mol^−1^ H_2_O) and the lowest for IL 3–5 (54.31 µmol CO_2_ mol^−1^ H_2_O) (Fig. 2d).

### Leaf thickness (LT) and minimum epidermal conductance (g_min_)

**Figure 3:**
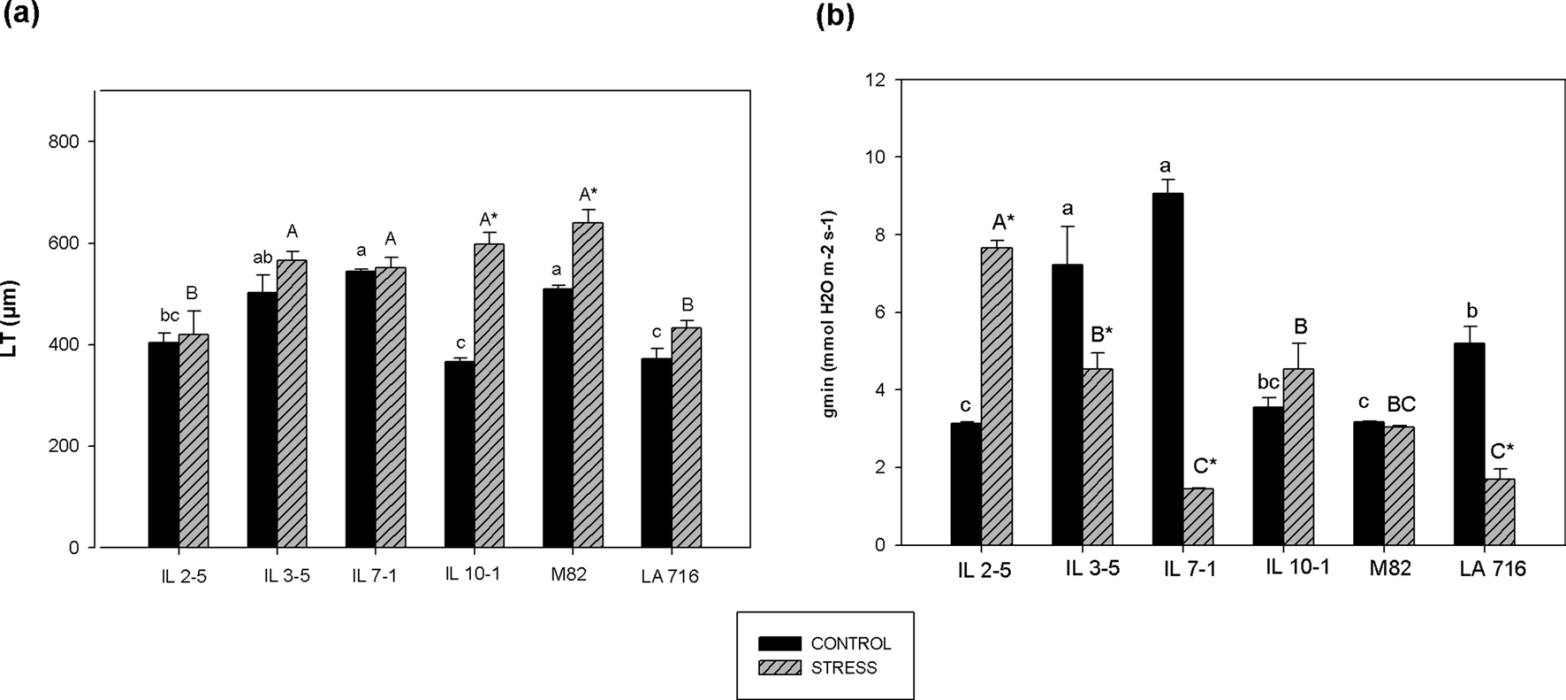
Leaf thickness (LT) (**a**) and minimum epidermal conductance (g_min_) (**b**) of tomato genotypes grown under two different water regimes (50 and 100% ASW), quantified on top third leaves, 60 days after the beginning of the stress treatment. Same lower- and upper-case letters indicate that the genotypes did not differ by the Tukey’s test (*p* > 0.05), in the control (100% ASW), and in the stress treatment (50% ASW), respectively. The asterisk (*) indicates statistical difference between control and stress treatment for the same genotype. Data are expressed as means ± standard error.

**Figure 4:**
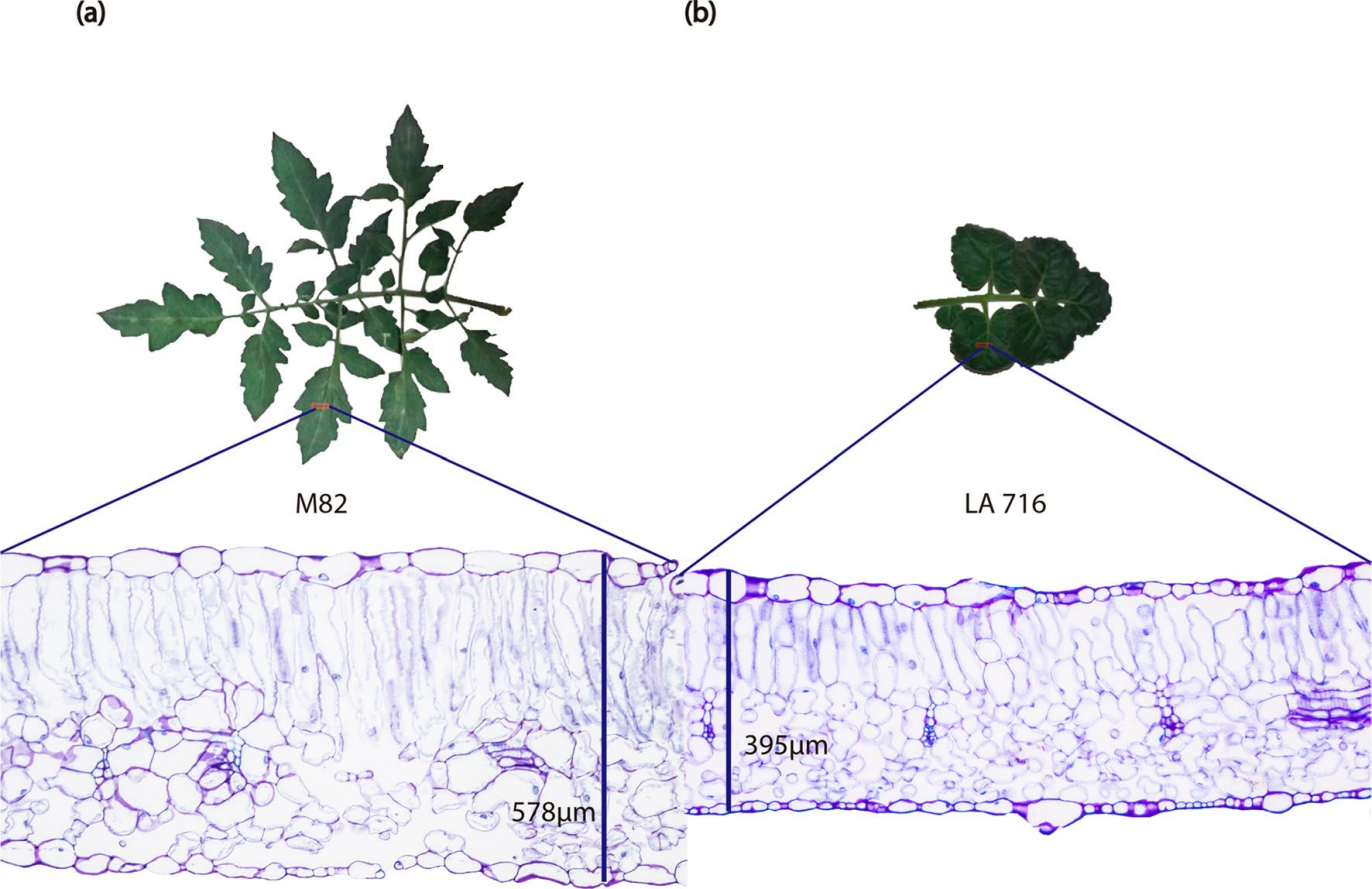
Leaf cross-section of M82 (**a**) and LA 716 (**b**), cultivated in 15L-pots inside a greenhouse, with soil available water kept at 100%. The leaflets were collected on top third leaves, 60 days after the beginning of the stress treatment (at fruit setting stage). Note that surprisingly at this growth stage M82 leaves were thicker that LA 716 leaves.

Leaf blade was, on average, 234 µm thicker in IL 10–1 plants grown under 50% than in IL 10–1 plants grown under 100% ASW. For M82, LT was also higher in plants grown under 50% ASW (Fig. 3a). This increase in LT was associated with an increase in spongy parenchyma and palisade parenchyma thickness in IL 10–1 plants and only spongy parenchyma thickness in M82 plants (data not shown). On the other genotypes, LT was not affected by the water regime (Fig. 3a). Unlike expected, LT was lower in LA 716 plants than in M82 plants (Figs. 3a, 4).

Low water regime (50% ASW) reduced g_min_ of IL 3–5, IL 7–1, and LA 716 while g_min_ of IL2-5 increased, and g_min_ of M82 and IL 10–1 stayed the same (*p* < 0.05). M82 showed low g_min_ on both irrigation treatments (Fig. 3b).

### Plant yield

**Figure 5:**
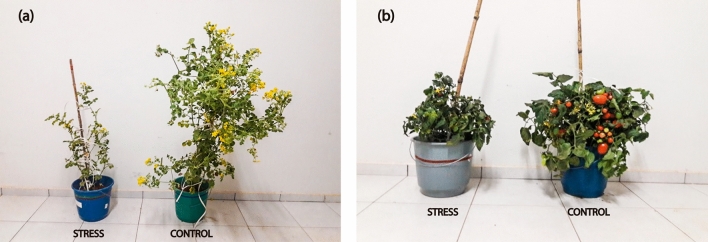
Visual differences on growth of LA 716 (**a**) and IL 10–1 (**b**) due to water supply during cultivation. Plants of the stress and control treatments were kept at 50 and 100% ASW, respectively.

Mean values of yield parameters are shown in Table 1. The low water regime imposed decreased fruit fresh weight (FW), fruit diameter (FD), and fruit length (FL) (*p* < 0.05), but it did not affect number of fruits per plant (FN) (*p* > 0.05). The highest FW at 100% ASW was detected for IL 3–5 (2.66 kg plant^−1^), and the lowest FW was detected for IL 2–5 and IL 10–1 (1.95 and 1.59 kg plant^−1^, respectively). FW did not vary between genotypes at 50% ASW (*p* > 0.05). Visual differences in plant growth as caused by the water supply provided on both control and stress treatments can be observed in Fig. 5. LA 716 control plants showed an astonishing growth pattern, reaching up to 180 cm in height with intense branching, in comparison to stress plants that were much shorter with fewer branches and leaves (Fig. 5).

**Figure 6:**
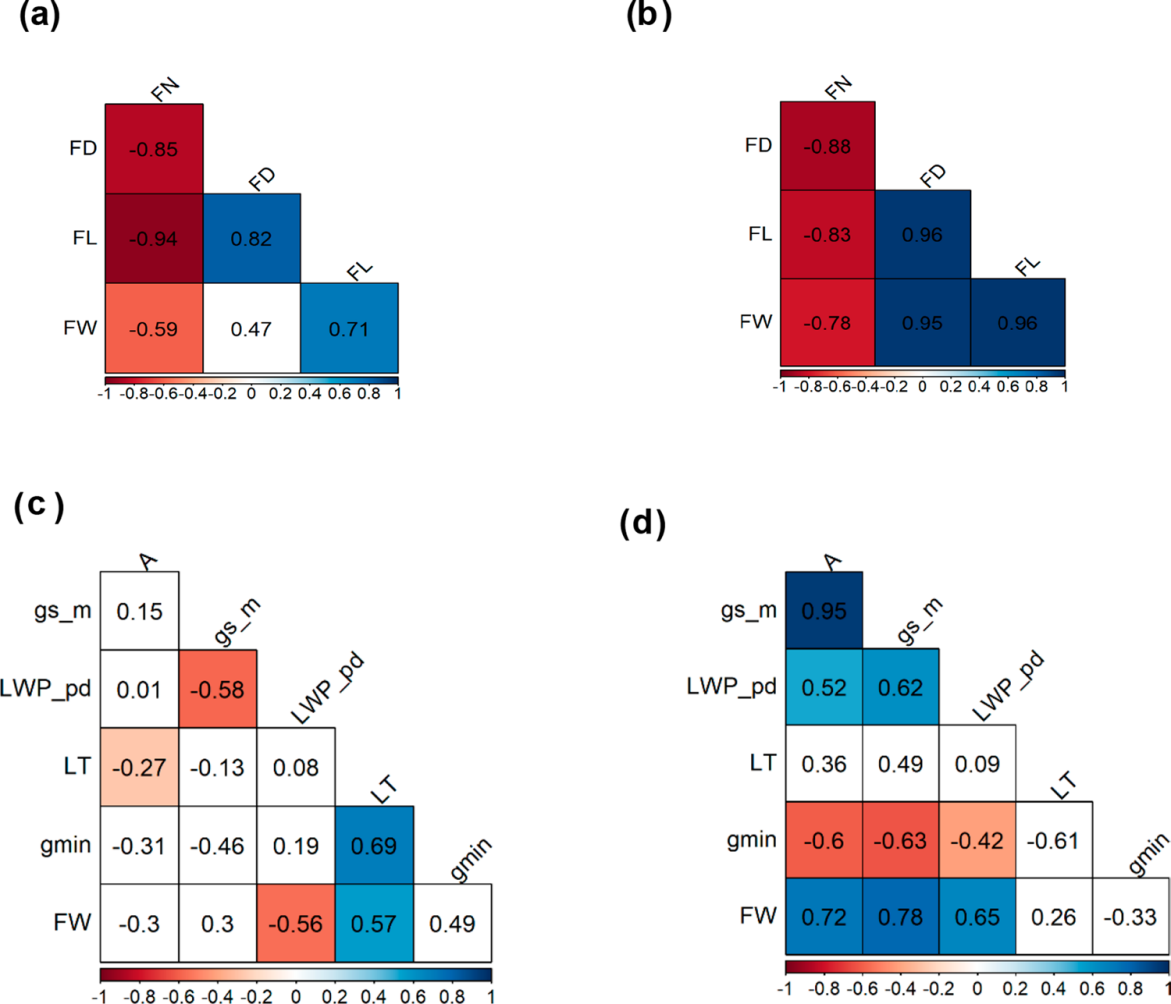
Pearson’s correlation matrix between plant yield parameters in the control (100% ASW) (**a**) and in the stress treatment (50% ASW) (**b**). FN = fruit number; FD = fruit diameter; FL = fruit length; FW = fruit fresh weight. Pearson’s correlation matrix between physiological and anatomical parameters in the control (**c**) and in the stress treatment (**d**). *A* = photosynthesis; gs_m = stomatal conductance in the morning (08:00–10:00); LWP_pd = leaf water potential at pre-dawn (03:00–05:00); LT = leaf thickness; g_min_ = minimum epidermal conductance. Blank squares mean that correlation was not significant by the t test (*p* > 0.05).

IL 2–5 and IL 10–1 yield higher FN (219 and 252 fruits per plant, respectively) when compared to the other studied genotypes. Both also showed lower values for FD, FL, and, as stated before, FW. It means that the genomic segments of LA 716 present on both ILs directly affect plant yield parameters making fruits unmarketable under water-limited conditions. Pearson’s correlation coefficients showed a strong linear association between FW and FN, FW and FD, and FW and FL on both water regimes (Fig. 6a,b). The greater FN, the lower FD and FL (Fig. 6a,b). The highest reduction on FD and FL was observed for IL 10–1 (47%) and IL 7–1 (43%), respectively, and the lowest reduction for both plant yield parameters was observed for IL 2–5 (30% for FD and 25% for FL).

**Figure 7:**
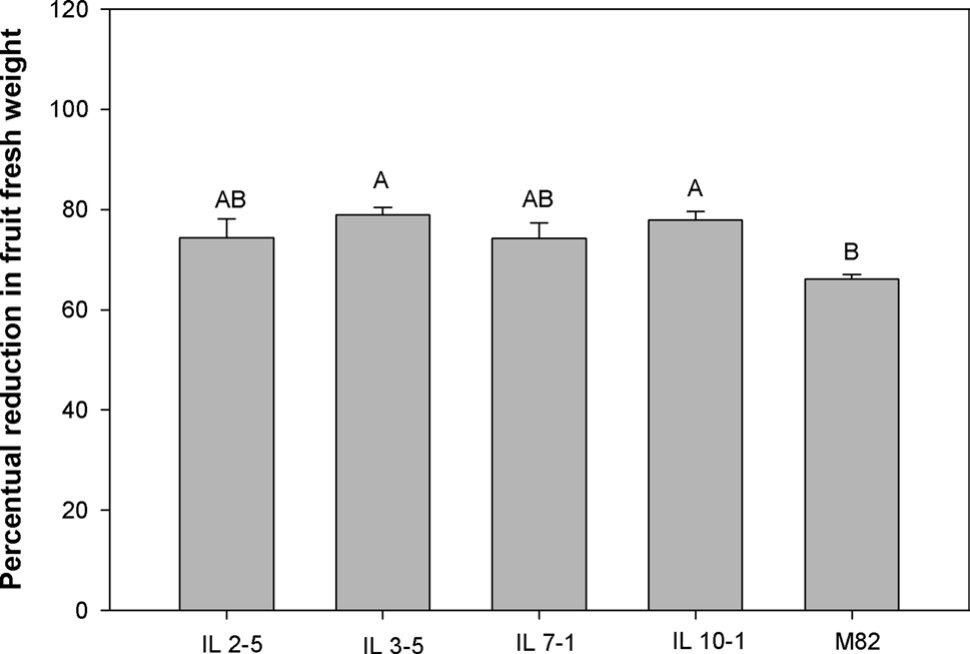
Percentual reduction in fruit fresh weight observed between stress (50% ASW) and control plants (100% ASW) from the same genotype. Means (± standard error) were compared by the Tukey’s test at significance level of 0.05.

Reduction of up to 79% in FW was observed for IL 3–5. M82 showed the lowest decrease in FW (66%), did not differing from FW reduction observed for IL 2–5 and IL 7–1 (Fig. 7).

### Correlation analyses

Percentage of yield loss negatively correlated with FW at 50% ASW (r = −0.77; *p* < 0.001). FW at 50% ASW also correlated with *A*, g_s_ in the morning, and Ψ_leaf_ at the pre-dawn evaluation (Fig. 6d).

LT of IL 10–1 was higher at 50% ASW than it was at 100%, but it did not result in increased FW at 50% ASW. Although positive correlation between LT and FW was significant on plants grown under 100% ASW (Fig. 6c), it was not significant on plants grown under 50% ASW (Fig. 6d).

FW did not differ between genotypes at 50% ASW and yet different behavior was observed for g_min_. g_min_ of IL 2–5 was 7.65 mmol m^−2^ s^−1^ on average while g_min_ of IL 7–1 was only 1.46 mmol m^−2^ s^−1^ on average. Alike LT, g_min_ positively correlated with FW at 100% ASW (r = 0.56; *p* < 0.05) but it did not correlate at 50% ASW (r = −0.48; *p* > 0.05).

## Discussion

Tomato is a high-water demand crop that produces fleshy fruits consumed both fresh or processed^25^. In order to be sold on the fresh market, tomato fruits should present a minimum size (e. g., in Brazil tomato fruits should have at least 55 mm in diameter^26^). Since 93–95% of tomato fruit composition is water^27^, production of marketable fruits without irrigation in locations where rainfall is scarce and irregular is impossible. Therefore, breeding for drought resistance in tomatoes aims at developing high-yielding genotypes, adequate in size, which requires a lower but regular water supply throughout the entire growing cycle. The introgression of drought-resistance traits, that are associated with high yield under low water regime, into elite germplasm is required on such breeding programs.

*Solanum* *pennellii* is a widely studied species regarding drought resistance in tomato^10^. Here, we investigated the anatomical and physiological behavior of *S. pennellii* accession LA 716, four ILs (derived from LA 716 × M82 cross) previously selected as drought-sensitive (IL 2–5 and IL 7–1) and drought resistant (IL 3–5 and IL 10–1) and the cultivar M82 under two water regimes (50 and 100% ASW) in order to understand their importance to tomato breeding for drought resistance.

Abiotic stresses are known for affecting since leaf functional traits and pigments of tomato plants until decreasing crop performance^28,29^. In this study, we also detected changes in leaf anatomy and gas exchange parameters when plants were subjected to limited water conditions, with contrasting behavior observed between the cultivated and the wild tomato species.

The ability of LA 716 to preserve water under low soil moisture content was clearly demonstrated by its high Ψ_leaf_ values. Ψ_leaf_ of *S. lycopersicum* during midday evaluation ranged from − 1.18 to − 1.48 MPa at 50% ASW, which indicates strong loss of leaf turgescence, while Ψ_leaf_ of S. *pennellii* plants ranged from − 0.5 and − 0.8 MPa. Similarly, Torrecillas et al.^9^ found Ψ_leaf_ values for *S. lycopersicum* close to − 1.0 MPa after 2 days of irrigation suspension while Ψ_leaf_ values of such magnitude were recorded for *S. pennellii* only at the 6th day.

This high leaf tissue hydration recorded for LA 716, even in the stress treatment, is likely associated with stomatal closure during critical day hours. At midday evaluation, g_s_ mean value for *S. lycopersicum* was 4.6 times higher than that found for *S. pennellii* in the control treatment and 5.8 times higher in the stress treatment. Similarly, Torrecillas et al.^9^, Rocha et al*.*^11^, and Egea et al*.*^28,30^ recorded inferior g_s_ values for *S. pennellii* under drought-stress conditions. As LA 716 showed high g_s_ in the control treatment, statistically identical to g_s_ values recorded for M82 (*p* < 0.05), low g_s_ values observed for LA 716 in the stress treatment are likely associated with stomatal closure rather than low stomatal density as suggested by Kebede et al*.*^14^ and Egea et al*.*^30^. In fact, Melo et al.^31^ found greater stomatal density in LA 716 plants in comparison to three *S. lycopersicum* genotypes.

Our results reveal differences in photosynthetic behavior between the studied genotypes. Water deficit decreases *A* via reduction in stomatal and mesophyll conductance to CO_2_, and photosynthetic metabolic potential^32–34^. However, reductions in *A* as affected by water deficit vary among plant species^35^, genotypes within a plant species^22^, and drought severity^30^.

A comprehensive study regarding gas exchange parameters using the Eshed and Zamir^15^ IL population was carried out by Silva et al*.*^22^. These authors found 16 QTLs with significant genotype effect for *A* (16 ILs had greater *A* than M82) suggesting a complex genetic architecture underlying photosynthesis within the IL population. In our study, all the four ILs showed *A* values statistically identical to M82 at 100% ASW which means that the introgressed genomic fragments present in the four ILs did not affect *A*. However when grown at 50% ASW, statistical differences were observed between M82 and IL 2–5, IL 3–5 and IL 10–1, indicating that the introgressed genomic fragments of IL 2–5, IL 3–5 and IL 10–1 caused a reduction in *A* under low water regimes. These findings point out different gene expression as plants are subjected to different water regimes, suggesting that QTL analyses for improved performance in drought-stress conditions, should be performed in plants previously exposed to water deficit.

As expected, we found that the low water regime applied in the stress treatment reduced Ci and *E* while increased T_leaf_, agreeing with several studies involving drought stress in tomato plants^30,36,37^. Since *E* and CO_2_ uptake occurs through stomata, a reduction on stomatal aperture would result on reduced Ci and *E*. As a consequence of reduced *E*, T_leaf_ increases since transpiration is an important mechanism of plant cooling^38^. Interestingly, we did not find statistical differences between genotypes for *E* and T_leaf_. A study conducted by Egea et al*.*^30^ , where M82 and LA 716 were exposed to water deficit stress through irrigation suspension, suggests that T_leaf_ and *E* vary within these tomato species only under severe stress conditions. While significant increase in T_leaf_ was observed in two days after water suspension, significant differences between M82 and LA 716 were seen after the third day without irrigation, with lower values recorded for the wild species^30^. For *E*, differences between species were found after four days of water suspension^30^.

WUE here increased as a result of drought-stress, especially for LA 716, but it did not correlate with FW in the stress treatment, which makes us question its real usefulness in plant breeding for drought resistance. WUE is defined as the biomass/water application ratio in agronomic terms or *A*/g_s_ in physiological terms. According to Blum^39^, most of the genotypic variation observed for this variable comes from changes in the denominator (water application or g_s_), not in the numerator (biomass or *A*). High values of WUE, therefore, are the result of less water use by plants (low denominator), which in turn reduces biomass and fruit yield under low water regimes.

The fact that M82 displayed lowest reduction in FW indicates that the chromosomal segments introgressed on all ILs studied here are not associated with drought resistance regarding plant yield maintenance under water-limited supply. Reduction in FW observed for the drought-resistant ILs (IL 3–5 and IL 10–1) was statistically higher than the reduction in FW observed for M82, suggesting that there is no correlation between drought resistance at seed germination and plant productive stages. Under mild and severe drought-induced conditions, Rigano^29^ found lower percentage of yield loss for IL 9–2–5 compared to M82 plants. This is an indicator of not only genetic variation for this trait within the IL population but also of the presence of genes positively regulating plant performance under water-limited conditions. Interestingly, IL 9–2–5 was also considered one of the most drought-sensitive genotypes together with IL 2–5 and IL 7–1 on our previous experiment performed at seed level (see Supplementary Fig. 1), suggesting once again the lack of association between drought resistance at seed germination and plant productive stages.

The significant reduction in plant yield parameters observed in this study and the fact that FW of IL 3–5 and IL 10–1 was statistically different from FW of M82 indicate that the stress treatment chosen was severe, but it was still useful for genotype screening. Imposing such severe stress to plants is interesting as it decreases the chance of selecting a genotype as resistant to drought when in fact this genotype is sensitive.

Although none of the studied ILs showed greater drought resistance than M82, our data shows a strong positive correlation between *A*, g_s_, and Ψ_leaf_ at 50% ASW. It is either through stomatal opening that the majority of water is lost and the CO_2_ enters the leaf tissue. Since tomatoes are C_3_ plants and have no CO_2_-concentrating mechanism, stomatal closure would undoubtedly result in reduced photosynthesis^40^. Under low Ψ_leaf_, the stomata close^40^ and there is no increase in leaf area^32^ with consequences to plant yield. Several studies show that high-yielding genotypes facing drought stress also present high g_s_ values^39,41,42^. Although in this study LA 716 showed higher water status throughout the day, this condition was maintained at the expense of CO_2_ uptake, which is not interesting for breeding purposes.

Of all adaptative mechanisms involved with *S. pennellii*’s survival in drought-prone environments^10^, lower g_min_^13^ and thicker leaves^24,43^ show potential use in tomato breeding programs for drought resistance due to their association with plant yield.

Increase in LT as affected by water deficit has been reported for several plant species^44–46^, including tomatoes^47^. In this study, LT of LA 716 was statistically identical on both water regimes which may be explained by the fact that LA 716 did not suffer from the limited water supply as much as the other genotypes, as can be confirmed by the Ψ_leaf_ values recorded, or even by the fact that this plant species evolved in a hot and dry region so that leaf thickness is no more affected by low soil water content.

Long-term exposure to water deficit seems to affect LT in a genotype-specific way. LT is a complex trait highly influenced by plant developmental stage, leaf age, and the growing environment^24^. Differently from what was observed for Coneva et al.^24^ and Muir et al*.*^23^, in this study, LT of M82 was higher than that of LA 716, probably because leaves were collected at fruit setting stage. In a study carried out by Coneva et al*.*^24^ M82 leaves were only 83 µm on average thinner than LA 716 leaves at vegetative stage. Such difference in leaf thickness between M82 and LA 716 is too small that could be easily surpassed at advanced plant developmental stages.

Increased LT has been associated with increased *A*^48,49^, which may lead to high plant yield at the end of the growing cycle. Thicker leaves accommodate greater concentration of photosynthetic apparatus per unit leaf area^48^. Also, increased LT is usually a result of an increment in mesophyll cell size. Large cells enable an increase in the surface area of chloroplasts facing intercellular airspaces, facilitating CO_2_ arrival at carboxylation sites^47^. Here, positive correlation between FW and LT was significant on tomato plants grown under 100% ASW, supporting the results found by Mvumi et al*.*^43^. Thicker leaves are also common traits on high-yielding genotypes of several plant species, including common beans^50^, rice^51^, and cashew^52^. Although we found strong correlation between FW and *A*, no correlation between LT and these two variables was observed at 50% ASW to our surprise.

Total epidermal conductance is the sum of both stomatal and cuticular conductance to water vapor^53^. When stomata are open, cuticular conductance (g_min_) is a negligible fraction of total epidermal water loss^54^. However, under water-deficit conditions, stomata tend to close and so water loss through the cuticle becomes significant^55^ and hence determines if a plant will reach or not an injurious leaf water content. It is common to observe low g_min_ values in desert-adapted plant species^56^. In fact, Bolger et al*.*^13^ observed three times more cuticular wax in *S. pennellii* plants than in *S. lycopersicum* plants, which consists mainly of long-chain alkanes, molecules that were previously suggested as responsible for increasing cuticle resistance to water flow^57–59^. Therefore, we expected lower g_min_ for LA 716 compared to M82, especially in the stress treatment, which did not happen here.

Studies investigating the relationship between cuticle composition and water permeability to water vapor are controversial. Whereas cuticular transpiration for Lee et al.^60^ was slower in specimens of *Camelina sativa* L. with high amounts of alkanes, Riederer and Schneider^61^ found no correlation (linear and non-linear) between cuticle water permeability and the amount of alkanes in citrus.

Low soil moisture promotes changes in cuticle composition resulting in reduced cuticular conductance to water vapor^62^, but yet it happens in a highly genotype-specific way^63^, which explains why IL-2–5, IL 10–1, and M-82 showed different behavior when exposed to water-limited conditions. According to Riederer and Schreiber^56^ and Duursma et al.^63^, the relationship between cuticular permeability to water vapor and its chemical composition and physical structure is complex and still not well understood despite innumerous attempts on that matter. Interestingly, g_min_ values of LA 716 and M82 plants grown under 50% ASW were statistically identical, suggesting that the ability of losing less water through leaf cuticle under water-limited supply some ILs may present was not necessarily inherited from the wild species.

Unlike water loss through stomata, water loss through leaf cuticle is not beneficial in terms of carbon assimilation. It happens because it is during stomatal transpiration that plants capture CO_2_ from the atmosphere. Genotypes presenting low g_min_ may have an advantage in terms of biomass production since more water is being diverted to stomatal transpiration. Therefore, we expected an inverse correlation between g_min_ and FW, even at low water regimes, which was not the case here. Likewise, Quisenberry et al.^64^ did not observe a correlation between g_min_ and cotton growth rate in non-irrigated cultivation. Yet, Jefferson et al.^65^ did not observe a relationship between g_min_ and leaf biomass production in alfalfa when cultivated under different water regimes. The truth is that the contribution of g_min_ to the total water loss by the leaves is minimal compared to stomatal transpiration^66^, which makes us wonder whether this contribution is relevant in terms of plant yield under low water regimes.

## Methods

### Plant materials and greenhouse experiment

To investigate the physiological and anatomical behavior of tomato genotypes exposed to low but regular water supply, four tomato introgression lines (ILs) IL 3–5 and IL 10–1, considered drought-resistant, and IL 7–1 and IL 2–5, considered drought-sensitive at seed level (Supplementary Fig. 1.) together with both parents, the processing tomato cultivar M82 and the *S. pennellii* accession LA 716, were grown under two distinct water regimes (50 and 100% available soil water) throughout the entire growing season.

The greenhouse experiment was conducted in the Research and Extension Farm Unit *Horta Velha* at Universidade Federal de Viçosa, Viçosa, MG, Brazil (20° 45′ 14′ S; 42° 52′ 53′ W; 648.74 m of altitude), from January to June 2018.

Seeds were sown in polystyrene trays of 128 cells each containing Tropstrato HT substrate (VIDA VERDE, Mogi Mirim, SP, BRA). Plants at the 3–4 true leaf stage were transplanted into 15L-pots (1 plant per pot) large enough to promote full plant growth, containing a mixture of soil, sand, and composted cow manure (3:1:1). Soil texture was classified as sandy clay (sand: 52.9%, silt: 4.5%, clay: 42.6%). Fertilizations as well as fungicide/insecticide applications were based on crop recommendations. Plants were tied up to bamboo sticks placed inside each pot to prevent them from falling.

The experiment was arranged in a 2 (water regimes) × 6 (genotypes) factorial scheme in a randomized block design with three replications. Each replication consisted of three plants arranged side by side.

### Irrigation management and calculation

The six genotypes were grown under two distinct water regimes: a stress treatment, where soil water content was kept at 50% available soil water (ASW), and a control treatment, where soil water content was kept at 100% ASW.

A soil sample was taken to estimate soil water retention curve parameters based on the Van Genuchten equation^67^ using the SWRC Fit software^68^.

All pots were filled with the same dry soil weight (W_ds_). Water weight (W_water_) at stress and control treatments in the first irrigation was determined by multiplying W_ds_ with the soil water content (kg kg^−1^) at soil water potentials of − 130 and − 33 kPa, respectively. To estimate water tension values corresponding to 50 and 100% ASW, we assume that field capacity and wilting point were reached at matric soil water potentials of − 33 and − 1500 kPa, respectively^69^.

Soil water content was then monitored by weighing each pot daily. On the following irrigations, W_water_ applied was determined as total pot weight (W_totalpot_) minus pot weight measured on the day. Total pot weight was calculated as follows: W_totalpot_ = W_rec_ + W_ds_ + W_water_ + W_plant_ + W_tutor_, where: W_rec_ = recipient weight, W_ds_ = dry soil weight, W_water_ = water weight, W_plant_ = plant weight, and M_tutor_ = tutor weight.

W_plant_ was determined by weighing same-age spare plants grown within the experiment. Every ten days one *S. lycopersicum* and one *S. pennellii* spare plant were harvested and weighed to adjust W_plant_ since both plant species have different growth speeds.

As genotypes were grown on 15L-pots within a greenhouse, we were able to ensure that plants only had access to the exact amount of water we provided. The stress treatment imposed here was severe for more accurate selection of drought-resistant materials.

### Measurements

All following measurements were taken on top third leaves, 60 days after the beginning of the stress treatment (at fruit setting stage), to ensure that all leaves from the stress treatment used on the analyses were formed during a period of long-term exposure to water deficit. It gives time to plants adjust to harsh conditions, and hence modifications (especially regarding leaf anatomy and leaf cuticular composition) are more likely to occur. Besides, fruit setting is a critical stage on which carbon assimilation is supposed to be intense to allow fruit growth.

### Leaf water potential (Ψ_leaf_)

Pre-dawn (3:00–5:00) and midday (12:00–14:00) Ψ_leaf_ measurements were performed according to the method of Scholander et al.^70^ using a pressure chamber. Tomato leaflets were excised and placed on the chamber within 20 s of collection. Ψ_leaf_ values for each repetition consisted of the mean value of two leaflets.

### Physiological traits

The physiological traits photosynthesis (*A*), stomatal conductance (g_s_), intercellular CO_2_ concentration (Ci), leaf temperature (T_leaf_), and transpiration rate (*E*), were measured in the morning (from 8:00 to 10:00) with an Infrared Gas Analyser (Irga LI-6400, LI-COR, USA). Leaves were placed in the cuvette to stabilize for at least two minutes before the first recording. Internal conditions of the leaf chamber were set at average temperature of 22 °C, external CO_2_ concentration of 400 μmol mol^−1^ air, flow rate of 300 μmol s^−1^, and saturating light of 1000 μmol photons m^−2^ s^−1^ with 10% blue light. *A* values for each repetition consisted of the mean of five recordings taken on 6 s-intervals each after leaf acclimation. Water use efficiency (WUE) was expressed by *A*/g_s_. To investigate the relationship between leaf tissue hydration (Ψ_leaf_) and g_s_ at critical day hours, we also measured g_s_ in the afternoon period (from 12:00 to 14:00).

### Leaf thickness (LT)

Central leaflets were collected and fixed in FAA_50_ solution for 24 h after which they were transferred to a 50% ethyl ethanol solution^71^. Segments of 4 × 6 mm in size, extracted from the central region of the leaflets (Fig. 4), were used to measure leaf thickness. These segments were dehydrated in ethylic series and embedded in methacrylate (LeicaHistoresin, LEICA, Chicago, IL, USA).

The methacrylate blocks were sectioned using an automatic rotative microtome (Leica RM 2155, UK). The 5-μm-thick sections were stained with Toluidine blue for 11 min^72^ and mounted in synthetic resin (Permount, FISHER SCIENTIFIC, Fair Lawn, NJ, USA).

Four 5-μm-thick sections per repetition were photographed (10 × objective) in a microscope (Olympus AX70) equipped with U-Photo system. The images were used to measure total leaf thickness (LT) using the Image-Pro Plus program. Three observations were made per image so that the value of each repetition consisted of the mean value of 12 observations.

### Minimum epidermal conductance (g_min_)

For g_min_ calculation, central leaflets (4 per repetition) were collected early in the morning and immediately packed in plastic bags. In the laboratory, the leaflets were left at room temperature for drying until their stomata were completely closed (approximately 1 h).

The petiole base was sealed with paraffin to ensure that all water loss occurred through the cuticle. Mass of leaflets was then determined 14 times at 20-min intervals with the aid of an analytical balance (minimum weight 0.0001 g).

Leaflets were scanned at the beginning and the end of the evaluations, and the mean leaf area determined using the Image-Pro Plus program. Only the last eight measurements (the latter portion of each desiccation curve) were used to calculate g_min_ since at the beginning of the evaluations the mass loss was non-linear with time. A standard equation (Arden Buck equation; 1996, Buck Research CR-1A User's Manual) was used to calculated g_min_ based on the changing mass, relative humidity, air temperature, leaf area, and vapor saturation pressure parameters using a spreadsheet tool^73^. Air temperature and RH were recorded every 5 min.

### Plant yield

Tomato fruits were harvested at fruit ripening stage. To observe the influence of low water regime on yield-related traits, we measured fruit fresh weight (FW) expressed in kg plant^-1^, number of fruits per plant (FN), average fruit diameter (FD) (cm), and average fruit length (cm) (FL). For plant yield parameters, each repetition consisted of the mean value of the three plants arranged side by side. FD and FL measurements were taken from all fruits by using a digital pachymeter for more precise results.

Plant yield was measured for all genotypes except LA 716 since it does not produce marketable fruits. Drought resistance here was expressed as percentual reduction in fruit fresh weight observed between stress and control plants from the same genotype, given by R_FW_ (%) = (FW_CONTROL_ − FW_STRESS_/FW_CONTROL_) × 100, where R_FW_ = percentual reduction in fruit fresh weight, FW_CONTROL_ = fruit fresh weight at 100% ASW and FW_STRESS_ = fruit fresh weight at 50% ASW.

### Statistical analyses

A two-way analysis of variance (ANOVA) was used to test the effect of the genotypes and the two water regimes as well as their interactions on each studied trait. When differences were significant by the F test, means were compared using the Tukey’s test at a significance level of 0.05. Pearson's linear correlation coefficients were also estimated and tested (t test, *p* < 0.05) between the studied traits.

## Conclusions

In this study, we confirmed the great ability to cope with drought-stress conditions that LA 716 exhibits by establishing a link with stomatal closure. We also observed that providing a limited-water supply to tomato plants throughout the growing cycle altered all physiological (Ψ_leaf_, *A*, g_s_, Ci, *E*, T_leaf_, WUE, g_min_) and anatomical (LT) traits studied depending on the genotype, which indicates that genotype selection for water-limited environments based on these traits must be performed under previous genotype exposure to reduced water supply. A QTL for enhanced FW under optimum irrigation conditions was found for the drought-resistant genotype at seed level IL 3–5, however, this QTL was not significant when plants were grown under water-limited conditions. As M82 showed lowest percentage of yield reduction, none of the four ILs studied here can be considered drought-resistant at plant productive stage. Interestingly, our data reveal that genotypes with lower percentage of yield reduction are the ones displaying high FW values under water-limited conditions (50% ASW), and as FW at 50% positively correlated with Ψ_leaf_, *A*, and g_s_, we suggest these traits as important selection criteria for breeding programs aiming at developing drought-resistant tomato cultivars. Selecting genotypes based on increased WUE, on the other hand, would lead to the selection of low-yielding genotypes. These results, together with the fact that QTLs for reduced g_s_ and *A* were found for IL 3–5 and IL 10–1 at 50% ASW indicate that these ILs have no use to our breeding program. *S. pennellii*’s most promising traits, LT and g_min_, showed to be extremely variable. Moreover, considering that LT and g_min_ are not associated with enhanced yield performance at limited-water supply and LA 716 has the tendency to close stomata when subjected to water-deficit conditions, our findings suggest that this wild accession has limited use in tomato breeding for drought resistance. In sum, this paper provides important insights for breeders regarding the successful selection of drought-resistant tomato genotypes.

## Supplementary information

Supplementary Information.

## Data Availability

You can contact the corresponding author F.D.D. for clarifications or request of materials at the e-mail address fran_dariva@hotmail.com.
