# Supplementary material for: Evaluation of anatomical and physiological traits of Solanum pennellii Cor. associated with plant yield in tomato plants under water-limited conditions
Source: Sci Rep. 2020 Sep 29;10:16052. doi: 10.1038/s41598-020-73004-4 (PMC7524713; doi:10.1038/s41598-020-73004-4)
Supplement: Supplementary file 1 — Supplementary Information. [file 41598_2020_73004_MOESM1_ESM.pdf]

Evaluation of anatomical and physiological traits of *Solanum pennellii* Cor. associated with plant yield in tomato plants under water-limited conditions

Françoise Dalprá Dariva[1*], Mariane Gonçalves Ferreira Copati[1], Herika Paula Pessoa[1], Flávia Maria Alves[1], Felipe de Oliveira Dias[1], Edgard Augusto de Toledo Picoli[2], Fernando França da Cunha[3], Carlos Nick Gomes[1]

[1]Department of Plant Science, Universidade Federal de Viçosa, Av. P.H. Rolfs, s/n, Campus Universitário, 36570-900, Viçosa, MG 36570-900, Brazil.
[2]Department of Plant Biology, Universidade Federal de Viçosa, Av. P.H. Rolfs, s/n, Campus Universitário, 36570-900, Viçosa, MG 36570-900, Brazil.
[3]Department of Agricultural Engineering, Universidade Federal de Viçosa, Av. P.H. Rolfs, s/n, Campus Universitário, 36570-900, Viçosa, MG 36570-900, Brazil.

*Corresponding author: fran_dariva@hotmail.com

Genotype selection

Before this experiment, we phenotyped 50 tomato genotypes under drought-induced conditions at germination and seedling stages: the cultivars M82 and Santa Clara, both considered drought-sensitive, the *S. pennellii* wild accession LA 716, and forty-seven *S. pennellii* introgression lines (IL's). Germination and seedling growth were evaluated under controlled and drought conditions. The experiment was arranged in a completely randomized design with four replications, being each replication consisted of an acrylic box containing 25 seeds. Several traits related to germination and seedling growth were measured. A multi-trait index based on factor analysis and genotype-ideotype distance (FAI-BLUP index) was used to accurately rank the 50 tomato genotypes according to their resistance to drought stress conditions. The complete ranking is shown in Supplementary Figure 1. below (data not published). LA 716 was ranked as the most drought-tolerant genotype, which indicates that the chosen methodology is adequate for genotype ranking. The most drought-resistant ILs were IL 3-5 and IL 10-1, and the most drought-sensitive were IL 7-1 and IL 2-5. Those IL's were then chosen to be further analyzed in the current study.

Supplementary Figure 1: Genotype ranking according to their drought resistance level at germination and early seedling growth stages using the FAI-BLUP index.

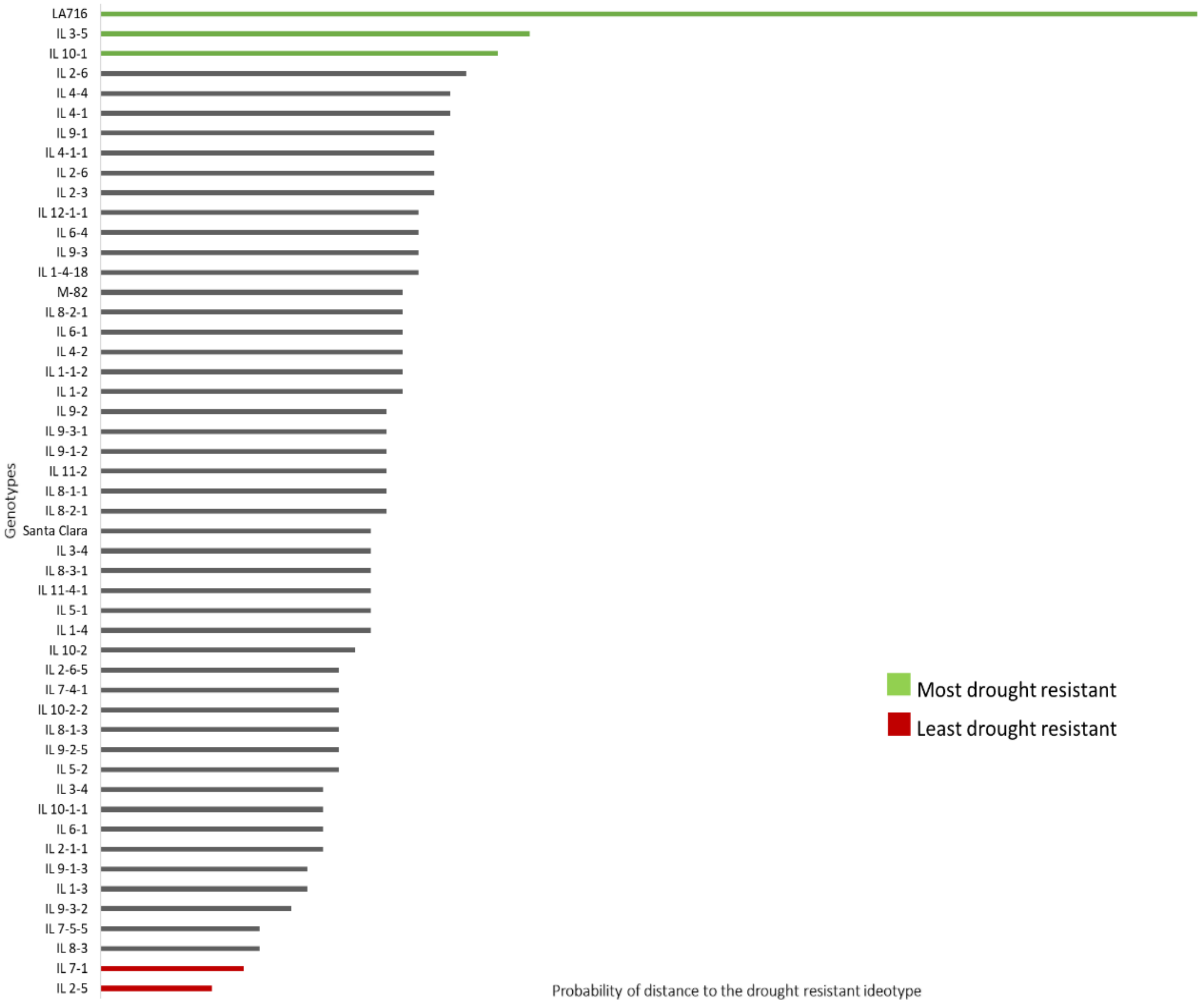

Supplementary Table 1. Intercellular CO_2 concentration (Ci), transpiration rate (*E*), and leaf temperature (T_{leaf}) of tomato genotypes grown under two different water regimes (50 and 100% ASW), quantified on top third leaves, 60 days after the beginning of the stress treatment. Genotype x water regime interaction was not significant by the F test (p>0.05) thus we studied both effects separately. Same lower-case letters indicate that the genotypes did not differ by the F (*E* and T_{leaf}) and the Tukey's test (Ci) (p>0.05). Same upper-case letters indicate that there was no statistical difference between control (100% ASW) and stress (50% ASW) treatment for the trait (p>0.05). Data are expressed as means ± standard error.

**GENOTYPE**	**Ci (µmol CO_2 mol^{-1})**		
	**CONTROL**	**STRESS**	**Genotype overall mean**
IL 2-5	306.23 ± 11.32	233.56 ± 10.88	269.91 ab
IL 3-5	320.27 ± 5.81	285.24 ± 14.11	302.75 a
IL 7-1	277.16 ± 18.79	245.15 ± 10.74	261.16 bc
IL 10-1	289.78 ± 3.39	252.28 ± 9.74	271.03 ab
M82	303.13 ± 19.10	263.53 ± 2.72	283.33 ab
LA 716	243.60 ± 4.00	206.39 ± 15.50	225.00 c
**Water regime overall mean**	290.04 A	247.69 B	

**GENOTYPE**	***E* (mmol H_2O m^{-2} s^{-1})**		
	**CONTROL**	**STRESS**	**Genotype overall mean**
IL 2-5	5.21 ± 0.26	1.51 ± 0.18	3.36 a
IL 3-5	5.45 ± 0.83	2.66 ± 0.62	4.06 a
IL 7-1	4.34 ± 1.18	3.09 ± 0.27	3.71 a
IL 10-1	4.77 ± 0.84	2.40 ± 0.61	3.59 a
M82	5.14 ± 0.49	3.33 ± 0.54	4.24 a
LA 716	5.63 ± 0.92	2.42 ± 0.14	4.03 a
**Water regime overall mean**	5.09 A	2.57 B	

**GENOTYPE**	**T_{leaf} (°C)**		
	**CONTROL**	**STRESS**	**Genotype overall mean**
IL 2-5	25.89 ± 2.22	27.46 ± 1.92	26.67 a
IL 3-5	26.95 ± 1.36	28.39 ± 1.94	27.67 a
IL 7-1	28.23 ± 0.90	28.66 ± 1.31	28.45 a
IL 10-1	26.39 ± 1.63	28.98 ± 1.41	27.68 a
M82	26.62 ± 0.90	26.90 ± 2.31	26.76 a
LA 716	27.55 ± 1.19	26.79 ± 2.53	27.17 a
**Water regime overall mean**	26.94 B	27.86 A	
